# Accumulation of IL‐17^+^ Vγ6^+^ γδ T cells in pregnant mice is not associated with spontaneous abortion

**DOI:** 10.1002/cti2.1008

**Published:** 2018-01-29

**Authors:** Barbara Polese, Virginie Gridelet, Sophie Perrier d'Hauterive, Chantal Renard, Carine Munaut, Henri Martens, David Vermijlen, Irah L King, Nathalie Jacobs, Vincent Geenen

**Affiliations:** ^1^ GIGA‐I^3^ Center of Immunoendocrinology GIGA Research Institute University of Liege Liege Belgium; ^2^ GIGA Laboratory of Tumor and Development Biology (LBTD) GIGA Research Institute University of Liege Liege Belgium; ^3^ Department of Pharmacotherapy and Pharmaceutics and Institute for Medical Immunology Université Libre de Bruxelles (ULB) Bruxelles Belgium; ^4^ Department of Microbiology and Immunology, Microbiome and Disease Tolerance Centre McGill University Montreal QC Canada; ^5^ GIGA‐I^3^ Laboratory of Cellular and Molecular Immunology GIGA Research Institute University of Liege Liege Belgium

**Keywords:** abortion, gamma delta T cells, interleukin‐17, pregnancy

## Abstract

**Introduction:**

Pregnancy is an immune paradox. While the immune system is required for embryo implantation, placental development and progression of gestation, excessive inflammation is associated with pregnancy failure. Similarly, the cytokine IL‐17A plays an important role in defence against extracellular pathogens, but its dysregulation can lead to pathogenic inflammation and tissue damage. Although expression of IL‐17 has been reported during pregnancy, the cellular source of this cytokine and its relevance to gestation are not clear.

**Objectives:**

Here we define the kinetics and cellular source of IL‐17A in the uterus during healthy and abortion‐prone murine pregnancy.

**Methods:**

The CBA/J x DBA/2J abortion‐prone mating was used and compared to CBA/J x BALB/c control mating.

**Results:**

We demonstrate that, irrespective of gestational health, the number of IL‐17‐producing cells peaks during midterm pregnancy and is largely derived from the γδ T‐cell lineage. We identify γδ T, Th17, CD8 T and NKT cells as the cellular source of IL‐17A in pregnant mice. Furthermore, we positively identify the Vγ6^+^ subset of uterine γδ T cells as the main producer of IL‐17A during both healthy pregnancy and abortive pregnancy.

**Conclusions:**

To conclude, the accumulation of uterine IL‐17^+^ innate‐like T cells appears not to adversely impact the developing foetus. Collectively, our results show that IL‐17^+^ γδ T cells are present in the uterus throughout the course of normal gestation and therefore may play an important role in healthy pregnancy.

## Introduction

During normal pregnancy, the maternal host co‐opts cells of the innate and adaptive immune system to accept the semi‐allograft foetus while remaining vigilant against pathogen invasion.^1^ Thus, pregnancy requires a tightly regulated balance between immune regulation and surveillance. Indeed, lack of immune control in the uterus can lead to excessive inflammation and pregnancy failure.^2^

Interleukin‐17A (IL‐17A) is an inflammatory cytokine that plays an important role in defence against extracellular pathogens at barrier sites such as the female reproductive tract but is also involved in various autoimmune reactions and graft rejection^3^ via sustained neutrophil recruitment and chronic inflammation.^4^ However, the contribution of IL‐17 to uterine health and the developing foetus is unclear. Early studies suggested that IL‐17 was associated with complications during pregnancy and infertility.^5^, ^6^, ^7^ However, more recent studies suggest a beneficial function for IL‐17 during pregnancy including the promotion of embryo implantation.^8^, ^9^, ^10^ Importantly, new biologics targeting IL‐17A and its receptor have shown clinical efficacy in the treatment of psoriatic disease and rheumatoid arthritis.^3^ Given the prevalence of these diseases in the female population and the requirement for lifelong use of these medications to prevent disease progression, understanding the impact of this cytokine pathway on pregnancy is vital for reproductive health.

Although first identified as a CD4^+^ T cell‐derived cytokine, recent studies examining nonlymphoid barrier tissues such as the lung, skin and gut indicate γδ T cells are a potent source of IL‐17A.^11^, ^12^, ^13^ γδ T cells are thymus‐derived and express a TCR (T‐cell receptor) composed of γ and δ subunits and exhibit less variability than classical αβ T cells.^14^ γδ T cells also display innate‐like properties whereby exposure to inflammatory cytokines is sufficient to induce robust cytokine secretion in the absence of specific antigen recognition. Although a previous study described the expansion of uterine IL‐17A‐producing γδ T cells during pregnancy, the kinetics of this response and whether this population is the dominant source of this cytokine during pregnancy was not examined.^15^ By comparing healthy pregnancy and abortion‐prone murine pregnancy, here we determine the kinetics of IL‐17A production in the uterus and identify Vγ6^+^ γδ T cells as the dominant source of this cytokine, regardless of the health of the developing foetus.

## Results and discussion

### Regulatory and IL‐17^+^ T cells are increased throughout normal and abortive gestation

**Figure 1:**
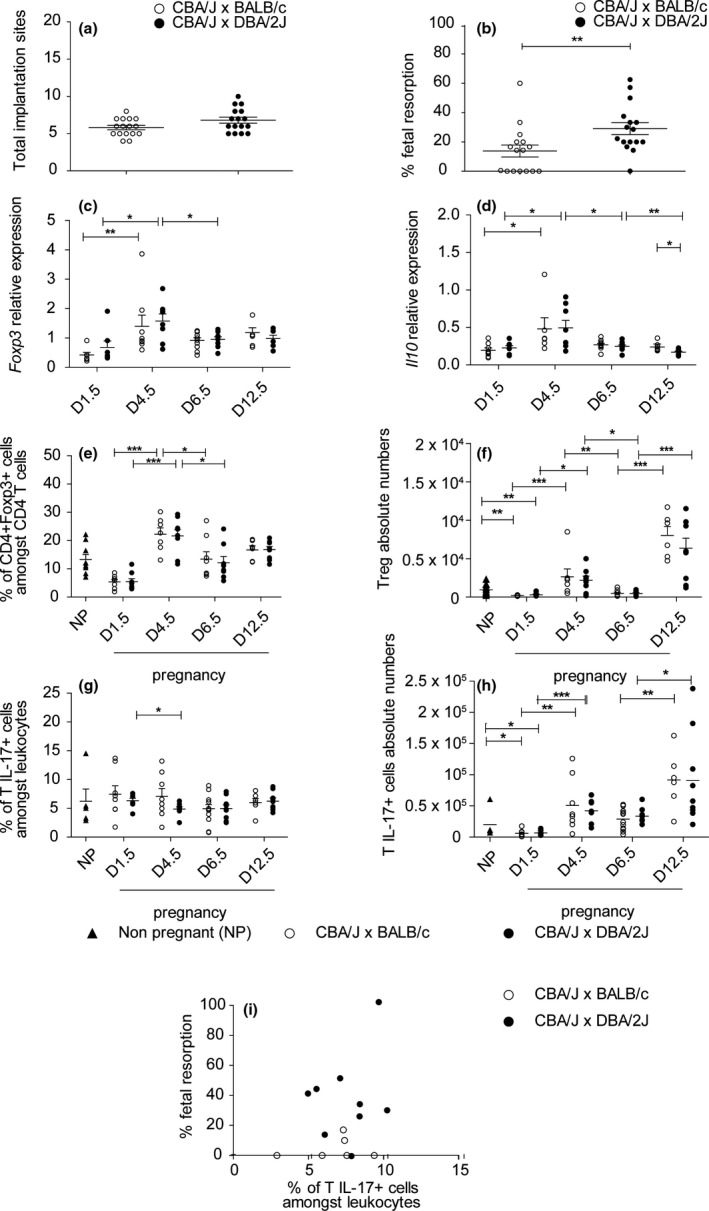
Regulatory and IL‐17^+^ T cells are increased throughout normal and abortive gestation. **(a)** Number of implanted foetuses counted at day 12.5 of pregnancy in uteri from DBA/2J‐ or BALB/c‐mated CBA/J females, *n* = 16 per group. **(b)** Rate of foetal resorption calculated at day 12.5 of pregnancy in uteri from DBA/2J‐ or BALB/c‐mated CBA/J females, *n* = 16 per group. **(c)** RT‐qPCR analysis of Foxp3 mRNA expression in uterine tissue of pregnant DBA/2J‐ or BALB/c‐mated CBA/J females on days of pregnancy 1.5, 4.5, 6.5, 12.5, *n* = 6–12. **(d)** RT‐qPCR analysis of Il10 mRNA expression in uterine tissue of pregnant DBA/2J‐ or BALB/c‐mated CBA/J females on days of pregnancy 1.5, 4.5, 6.5, 12.5, *n* = 6–12. **(e)** Percentage of Treg (CD4^+^ Foxp3^+^ cells) amongst CD3^+^ CD4^+^ cells in uteri from pregnant DBA/2J‐ or BALB/c‐mated CBA/J females on days of pregnancy 1.5, 4.5, 6.5, 12.5 and from nonpregnant BALB/c females, *n* = 6–12 per group. **(f)** Absolute Treg cell numbers in uteri from pregnant DBA/2J‐ or BALB/c‐mated CBA/J females on days of pregnancy 1.5, 4.5, 6.5, 12.5 and from nonpregnant BALB/c females, *n* = 6–12 per group. **(g)** Percentage of IL‐17A‐producing T cells (CD3^+^ IL‐17A^+^ cells amongst CD45^+^ cells) in uteri from pregnant DBA/2J‐ or BALB/c‐mated CBA/J females on days of pregnancy 1.5, 4.5, 6.5, 12.5 and from nonpregnant BALB/c females at oestrus phase, *n* = 6–12 per group. **(h)** Absolute cell numbers of IL‐17A‐producing T cells (CD3^+^ IL‐17a^+^ cells amongst CD45^+^ cells) in uteri from pregnant DBA/2J‐ or BALB/c‐mated CBA/J females on days of pregnancy 1.5, 4.5, 6.5, 12.5 and from nonpregnant BALB/c females, *n* = 5–12 per group. **(i)** Correlation between the foetal resorption rate and IL‐17A‐producing cells proportion (IL‐17A^+^ cells amongst CD45^+^) in uteri of pregnant DBA/2J‐ or BALB/c‐mated CBA/J females on day 12.5 of pregnancy, *n* = 14. Results are shown as mean ± SEM and are representative of at least three experiments. Mann–Whitney and Kruskal–Wallis tests were used. *P <* *0.05, **P <* *0.01, ***P <* *0.001. ▲ Nonpregnant, ○ CBA/J x BALB/c, ● CBA/J x DBA/2.

In order to study IL‐17A‐producing cells in normal conditions as well as in abortive gestation, we established a mouse model of abortion‐prone pregnancy. In this model, CBA/J x DBA/2J mating (called ‘abortive mating’) results in an increased frequency of rejected foetuses than CBA/J x BALB/c control breeders (called ‘control mating’).^16^ This model was tested in our animal care facility and, as expected, implantation rates were not significantly different between groups, while a significantly higher resorption rate was observed in CBA/J x DBA/2J mating (Figure 1a, b). Implantation and abortion rates were similar for all groups of this study. To further validate our model, we assessed the kinetics of Foxp3^+^ T regulatory (Treg) cell accumulation, an immunoregulatory lymphocyte subset that plays a critical role at the maternal–foetal interface.^5^ In order to maximise cell yield and to provide a general overview of the entire uterine immune cell compartment during pregnancy, we used entire uterine tissue. Consistent with previous studies,^17^, ^18^ Treg‐associated genes *Foxp3* and *Il10* expression was significantly increased during the implantation period, corresponding to day 4.5 (Figure 1c, d). In addition, the proportion as well as absolute cell number of Treg cells were significantly increased in the uterus throughout pregnancy and particularly at the implantation period and midgestation (day 12.5) (Figure 1e, f). However, there were no significant differences between healthy and abortive matings. This result is discordant with a study showing decreased Treg proportion in abortive mating using a different gating strategy^19^ but in accordance with another paper using the same gating strategy as in our studies.^20^ Since Treg have been shown to be particularly important for implantation rather than midgestation,^18^ and our model does not exhibit defective implantation, it is conceivable that Treg are not key players at this latter stage of pregnancy.

We next determined the kinetics of IL‐17A‐producing cells during the course of gestation. While no significant variation was found in the proportion of uterine IL‐17^+^ leucocytes (likely due to a recruitment of other cell types), the absolute number increased at implantation as well as during midgestation compared to nonpregnant controls, the latter time point corresponding to the time of visible embryo rejection. The increase during the embryo resorption period suggested that IL‐17^+^ cells could be linked with abortion. However, we found no significant correlation between the proportion or number of IL‐17A‐producing cells and abortion rates (Figure 1g–i). Similarly, the analysis of decidua samples of viable and resorbed foetus revealed no difference for IL‐17A‐producing cells (data not shown). These data suggest that IL‐17A is not a key mediator of abortion in this model, an unexpected result given the prominent concept that IL‐17‐producing cells are thought to be detrimental for pregnancy. Indeed, Th17 cells are increased in infertile women,^5^ and IL‐17A injection can lead to abortion in the abortion‐prone model.^7^, ^21^ However, increasing evidence suggests that Th17 cells may not necessarily be deleterious for gestation. For example, decidual Th17 cells are nonpathogenic and could be beneficial when they also produce IL‐4.^9^ Moreover, IL‐17A can promote trophoblast invasion and proliferation as well as progesterone secretion, which are essential for gestation.^8^, ^22^

### γδ T cells are the main producers of IL‐17A in pregnant mice

**Figure 2:**
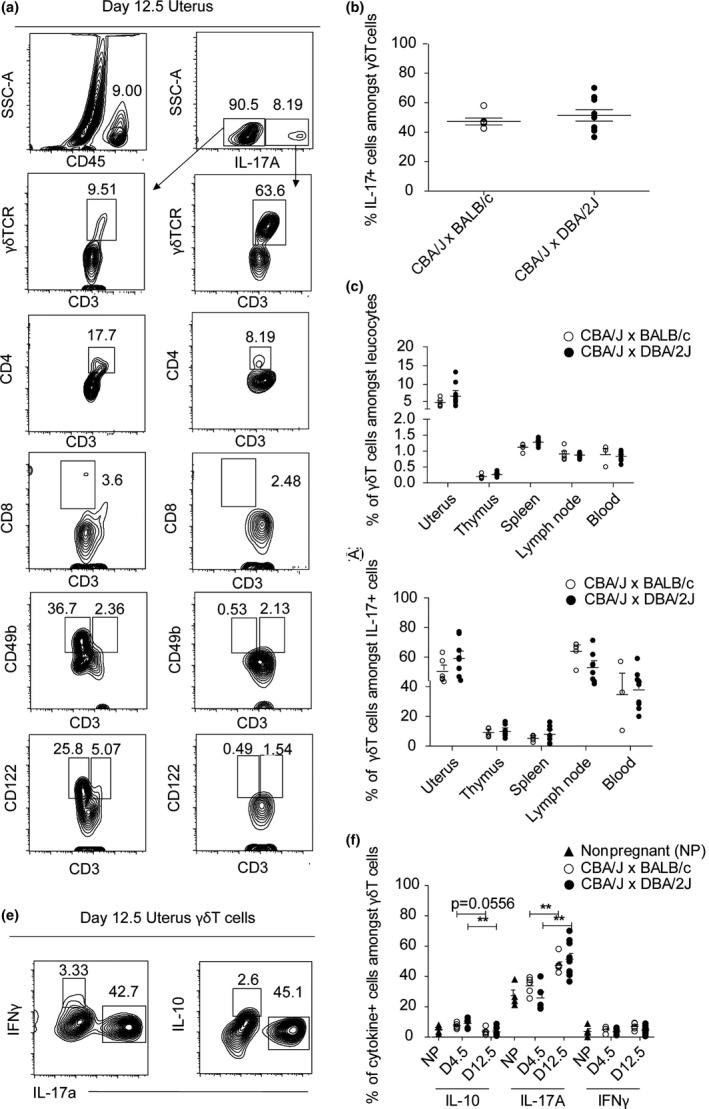
γδ T cells are the main producers of IL‐17A in pregnant mice. **(a)** Frequency of IL‐17A‐producing cells in the uterus of a representative pregnant BALB/c‐mated CBA/J female at day 12.5 of pregnancy. Numbers indicate frequency of the gated population. On the left, corresponding positive staining controls from CD45^+^ IL‐17A cells. On the right, IL‐17A^+^ cells. γδ TCR ^+^ CD3^+^ were used for γδ T cells. CD4 and CD8 were used in addition to CD3 cells to identify CD4 and CD8 T cells. CD49b and CD122 were used to identify NK cells, in addition to CD3 for NKT cells. **(b)** Percentage of IL‐17^+^ cells amongst γδ T cells in uteri of pregnant DBA/2J‐ or BALB/c‐mated CBA/J females on days 12.5 of pregnancy and of nonpregnant CBA/J females, *n* = 5–8 per group **(c)** Percentage of γδ T cells amongst CD45^+^ cells in various organs of pregnant DBA/2J‐ or BALB/c‐mated CBA/J females on day of 12.5 of pregnancy, *n* = 5–8 per group. **(d)** Percentage of IL‐17A‐producing γδ T cells (CD3^+^ γδTCR ^+^ cells amongst IL‐17^+^ cells) in various organs of pregnant DBA/2J‐ or BALB/c‐mated CBA/J females on day of 12.5 of pregnancy, *n* = 5–8 per group. **(e)** Frequency of cytokine‐producing‐γδ T cells (cytokine+ amongst CD3^+^ γδTCR ^+^ cells) in stimulated (with PMA and ionomycin) total uterine single cells of a representative pregnant BALB/c‐mated CBA/J female at day 12.5 of pregnancy. **(f)** Percentage of cytokine‐producing γδ T cells (IL‐10^+^, IL‐17^+^ and IFNγ^+^ cells amongst CD3^+^ γδTCR ^+^ cells) in uteri of pregnant DBA/2J‐ or BALB/c‐mated CBA/J females on days 4.5 and 12.5 of pregnancy and of nonpregnant CBA/J females, *n* = 3–10 per group. Results are shown as mean ± SEM and are representative of at least three experiments. Mann–Whitney test was used. *P < 0.05, **P < 0.01, ***P < 0.001. ▲ Nonpregnant, ○ CBA/J x BALB/c, ● CBA/J x DBA/2J.

We next sought to determine the cellular source of IL‐17A in pregnant mice (Figure 2a). As the peak of the IL‐17 response was at day 12.5 of gestation, we examined the phenotype of IL‐17^+^ cells at that time point (Figure 1f). Consistent with previous studies examining IL‐17A during pregnancy, a small population of IL‐17‐producing CD4^+^ αβ T (Th17) cells was detected (Figure 2a).^5^ Unexpectedly, however, the majority of IL‐17^+^ T cells were γδ TCR^+^ (Figure 2a). Nevertheless, the frequency of IL‐17^+^ γδ T cells did not differ between healthy and abortion‐prone females (Figure 2b). In addition, a small population of CD3^−^IL‐17A^+^ cells was observed (likely type 3 innate lymphoid cells, not depicted).^23^

The proportion of γδ T cells was remarkably higher in the uterus when compared to other lymphoid organs (Figure 2c). In addition, we noticed that γδ T cells were the main producers of IL‐17A in the uterus as well as the uterus‐draining inguinal lymph nodes at midgestation, yet were not different between groups of pregnant females (Figure 2d). As some reports indicated that γδ T cells may be protective during pregnancy,^24^, ^25^ we examined whether this cell subset also produced IL‐10 and IFNγ, two cytokines associated with gestational health.^26^, ^27^ However, few γδ T cells were positive for IL‐10 or IFNγ production in either group (Figure 2e, f).

### Vγ6^+^ T cells are the main subpopulation of γδ T cells and dominant producer of IL‐17A in pregnant mice

**Figure 3:**
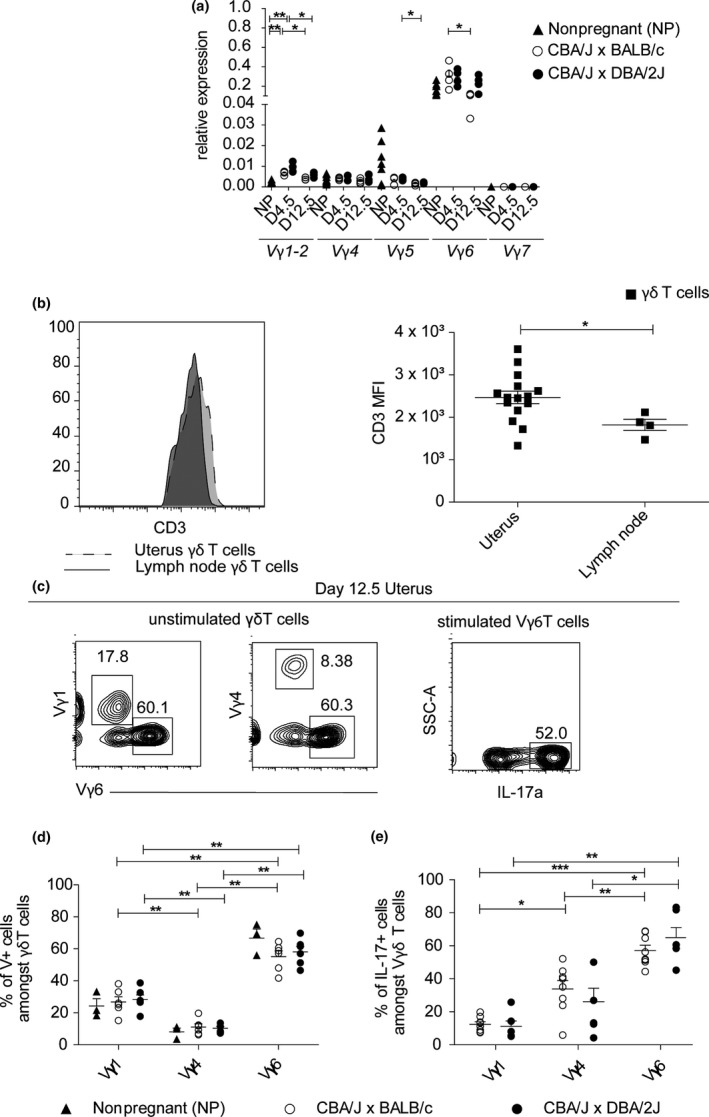
Vγ6^+^ T cells are the main subpopulation of γδ T cells and dominant producer of IL‐17A in pregnant mice. **(a)** RT‐qPCR analysis of TCRγ subunit expression in uterine tissue from nonpregnant CBA/J females and pregnant DBA/2J‐ or BALB/c‐mated CBA/J females on days 4.5 or 12.5 of pregnancy, *n* = 5–6 per group. **(b)** CD3 expression on γδ T cells from uterus and lymph nodes at day 12.5 of gestation. On the left, histogram showing a modal distribution of CD3 expression by uterine and lymph nodes total γδ T cells. On the right, CD3 MFI by uterine and lymph nodes total γδ T cells. **(c)** Frequency of γδ T subtypes in unstimulated γδ T cells and frequency of IL‐17A^+^‐producing‐stimulated (with PMA and ionomycin) Vγ6 γδ T cells, from the total uterine single cells suspension of a representative pregnant BALB/c‐mated CBA/J female at day 12.5 of pregnancy. Numbers indicate frequency of gated population. **(d)** Percentage of γδ T subtypes (Vγ^+^ cells amongst CD3^+^ γδTCR ^+^ cells) in uterus of pregnant DBA/2J‐ or BALB/c‐mated CBA/J females on day 12.5 of pregnancy and of nonpregnant CBA/J females, *n* = 3–6 per group. **(e)** Percentage of IL‐17A‐producing γδ T subtypes (IL‐17A^+^ cells amongst CD3^+^ γδTCR+Vγ^+^ cells) in the uteri of pregnant DBA/2J‐ or BALB/c‐mated CBA/J females on day 12.5 of pregnancy, *n* = 4–7 per group. Results are shown as mean ± SEM and are representative of at least three experiments. Mann–Whitney test was used. *P < 0.05, **P < 0.01, ***P < 0.001 ▲ Nonpregnant, ○ CBA/J x BALB/c, ● CBA/J x DBA/2J.

Previous work has suggested that uterine γδ T cells have limited TCR diversity and predominantly express the Vγ6 subunit. However, these conclusions were only based on transcriptional studies^28^ or by exclusion of other surface Vγ subunit expression.^15^ To positively determine the dominant subset of γδ T cells in the uterus during pregnancy, we used qPCR and flow cytometry‐based assays. Consistent with previous gene expression studies,^28^ RT‐qPCR analysis revealed an enrichment for Vγ6 mRNA. Furthermore, the predominance of this Vγ subunit remained stable throughout pregnancy (Figure 3a). To determine whether this pattern of Vγ expression translated to surface protein expression, we performed flow cytometry using antibodies specific for Vγ1, Vγ4 and Vγ6 TCR subunits. Uterine γδ T cells expressed higher levels of CD3 compared to those isolated from lymph nodes and, consistent with our transcriptional analyses, Vγ6^+^ cells were the dominant subtype of γδ T cells found in pregnant mice (Figure 3b, c). Notably, the percentage of uterine Vγ6^+^ T cells at midgestation was not different between healthy and abortion‐prone dames (Figure 3d).

Although a recent study by Pinget *et al*. suggested that Vγ6^+^ T cells were an important source of IL‐17A, they used high expression of CD3 (i.e. CD3^++^) as a proxy for Vγ6 expression but did not positively identify these cells.^15^, ^29^ To confirm that uterine Vγ6^+^ T cells were the dominant source of IL‐17A, we combined Vγ subunit staining with intracellular cytokine staining. Indeed, the percentage of IL‐17A^+^ Vγ6^+^ cells was significantly greater than Vγ4^+^ or Vγ1^+^ T cells (Figure 3d). Again, however, the percentage of IL‐17A^+^ Vγ6^+^ T cells was similar irrespective of gestational health (Figure 3e).

Regulation of IL‐17A production at barrier sites is critical for immune homoeostasis and health. Our study is the first to report the kinetics of IL‐17A^+^ T cells in the uterus over the course of both normal pregnancy and abortion‐prone murine pregnancy. In addition, we have definitively determined that Vγ6^+^ T cells are the dominant source of IL‐17A in pregnant mice. The frequency of total IL‐17^+^ cells, IL‐17^+^ γδ T and IL‐17A^+^ Vγ6^+^ T cells did not differ between healthy and abortion‐prone females. These data show that IL‐17A is not a key mediator of abortion in this model. As the source and number of uterine IL‐17^+^ γδ T cells occur independent of gestational health, our data suggest an alternative role for IL‐17 production during pregnancy that may have evolved to combat pathogenic challenge during the course of gestation rather than directly affecting foetal health. Given the recent development of therapies that target the IL‐17 pathway, it will be important to further investigate how modulating the function of this cytokine may impact the uterine microenvironment and the complex immunoregulatory pathways operating during human pregnancy.

## Methods

### Mice

CBA/J female, BALB/c and DBA/2J male mice were obtained from Charles River (France) and housed in SPF (specific pathogen free) conditions and used at 8–12 weeks of age. Mating was performed in trios, and the presence of a vaginal plug was assessed every morning and its appearance was considered as day 0.5. Pregnant females were then separated from the male and sacrificed at days 1.5, 4.5, 6.5 or 12.5 of pregnancy. At day 12.5, normal and naturally aborted foetuses were counted for every group of mice. Inguinal lymph nodes, thymi and spleens were collected before cell isolation. Entire uteri were also taken and placenta and its associated membranes were carefully removed before uteri were opened longitudinally for cell isolation. All experiments were approved by the Animal Ethics Committee of the University of Liege (permit N° 1373).

### Flow cytometry

Single‐cell suspensions were prepared by mechanical (lymph nodes, thymi and spleens tissues were crushed with a 5‐ml syringe plunger (VWR, USA)) or enzymatic disruption (uterine tissues were digested during 2 h at 37°C with 1 mg ml^−1^ collagenase A and 10 μg ml^−1^ DNAse I solution in RPMI medium). Cells dedicated to measurement of cytokine production by flow cytometry were first stimulated with 100 ng ml^−1^ PMA (phorbol myristate acetate) and 1 μg ml^−1^ ionomycin (Sigma, USA) for 3 h at 37°C with 5% CO_2_. For analysis of Treg cells, the following antibodies (Ab) were used as follows: anti‐mouse CD45.2 (104), CD3e (145‐2C11), CD4 (RM4‐5), purchased from BD Biosciences (Becton Dickinson, USA) and anti‐mouse Foxp3 (FJK‐16s) from eBiosciences (Affymetrix, USA). Transcription factor buffer set from BD Pharmingen (Becton Dickinson, USA) was used with manufacturer's protocol. For the study of cytokine‐producing cells and γδ T cells, the following antibodies (Ab) were used as follows: anti‐mouse CD45.2 (104), CD45 (30‐F11), CD3e (500A2), CD8 (53‐6.7), IL‐10 (JES5‐16E3) and IFN‐γ (XMG1.2) from BD Biosciences and γδ TCR (eBioGL3), CD122 (TM‐b1), TCR Vγ2 (UC3‐10A6), IL‐17A (eBio17B7) and anti‐rat IgM (RM‐7B4) from eBiosciences (Affymetrix, USA), anti‐mouse TCR Vγ1.1/Cr4 (2.11) from Biolegend (USA). Anti‐mouse IgM Vγ6 antibody (clone 17D1) was produced and kindly provided by Immo Prinz (Institute of Immunology, Hannover Medical School, Hannover, Germany). The BD Cytofix/Cytoperm Fixation/Permeabilization kit from BD Biosciences was used for intracellular cytokine staining according to manufacturer's instructions. FACS (fluorescence‐activated cell sorting) Verse machine with FACS Suite software (BD Biosciences) were used for flow cytometry. FCS files were analysed using Flow Jo V10 software. Gating strategy for T analysis was adapted from Rei *et al*.^30^

### RNA extraction

Lymph nodes and thymic (as control) and uterine tissue samples were removed and kept in RNA Later (Qiagen, Germany) before RNA extraction. Tissues were disrupted with MagNA Lyser Green Beads (Roche, Germany) in a MagNA Lyser instrument (Roche, Germany), and total RNA was extracted using the RNeasy Plus Mini Kit (Qiagen, USA) according to manufacturer's instructions.

### RT‐qPCR

For RT‐qPCR, the Transcriptor First Strand cDNA Synthesis Kit (Roche, Germany) and the Takyon No Rox SYBR MasterMix (Eurogentec, Belgium) were used with prevalidated specific primers purchased from Eurogentec (Belgium). RT‐qPCR products were sequenced at the Sequencing platform of the GIGA Genomics Facility by Sanger method to ensure correct sequence amplification. Relative gene expression was determined using the 2^−ΔΔCt method. Hypoxanthine‐guanine phosphoribosyltransferase, succinate dehydrogenase complex flavoprotein subunit A and β‐actin were used as housekeeping genes, and nonpregnant mice were used as untreated controls.

### Statistical analysis

Statistical analyses were performed with Prism 4.0 software (GraphPad). Normal distribution was first determined with Shapiro–Wilk normality test. When data were normally distributed, unpaired *t* Student tests were used. Mann–Whitney test was applied when normality was not observed. Parametric one‐way analysis of variance (ANOVA) or nonparametric Kruskal–Wallis tests were used to evaluate the data variance throughout pregnancy. Mean differences were considered significant when *P* < 0.05. All results are shown as mean ± SEM.
